# Supplementary material for: Malignant Hyperthermia in PICU—From Diagnosis to Treatment in the Light of Up-to-Date Knowledge
Source: Children (Basel). 2022 Nov 4;9(11):1692. doi: 10.3390/children9111692 (PMC9717737; doi:10.3390/children9111692)
Supplement: Supplementary file 1 [file children-09-01692-s001.zip › children-1922123-Supplementary.pdf]

Table S1: The example of possible initial dantrolene bolus preparation in children

Initial dantrolene bolus	
**Actual body weight (kg)**	**n. of vials* needed, total amount of solution** (mL) per bolus, n. of syringes**
≤1kg	1 vial, 10 mL from the syringe
1-2 kg	1 vial, 15 mL from the syringe
2-3 kg	1 vial, 20 mL from the syringe
3-5 kg	1 vial, 30 mL from the syringe
5-7 kg	1 vial, 45 mL from the syringe
7-10 kg	1 vial, 60 mL = whole syringe
10-15 kg	2 vials, 90 mL = 1,5 syringes
15-20 kg	2 vials, 120 mL = 2 syringes
20-25 kg	3 vials, 150 mL = 2,5 syringes
25-30 kg	3 vials, 180 mL = 3 syringes
30-35 kg	4 vials, 240 mL = 4 syringes
35-40 kg	5 vials, 300 mL = 5 syringes
45-50 kg	6 vials, 360 mL = 6 syringes
50-55 kg	7 vials, 420 mL = 7 syringes
55-60 kg	8 vials, 480 mL = 8 syringes
60-70 kg	9 vials, 540 mL = 9 syringes
70-80 kg	10 vials, 600 mL = 10 syringes
80-90 kg	11 vials, 660 mL = 11 syringes
90-100 kg	12 vials, 720 mL = 12 syringes
100-110 kg	13 vials, 780 mL = 13 syringes
110-120 kg	14 vials, 840 mL = 14 syringes
> 120 kg	15 vials, 900 mL = 15 syringes
*1 vial of dantrolene contains 20 mg of dantrolene sodium; **the 20 mg vial must be reconstituted with 60 mL sterile water, use a 50 mL syringe filled with up to 60 mL	
